# Engagement With Stop Smoking Services After Referral or Signposting: A Mixed-Methods Study

**DOI:** 10.1093/ntr/ntae159

**Published:** 2024-07-03

**Authors:** Ian Pope, Simrun Rashid, Hassan Iqbal, Pippa Belderson, Emma Ward, Lucy Clark, Tom Conway, Susan Stirling, Allan Clark, Sanjay Agrawal, Linda Bauld, Caitlin Notley

**Affiliations:** Norwich Medical School, University of East Anglia, Norwich, UK; Norwich Medical School, University of East Anglia, Norwich, UK; Norwich Medical School, University of East Anglia, Norwich, UK; Norwich Medical School, University of East Anglia, Norwich, UK; Norwich Medical School, University of East Anglia, Norwich, UK; Norwich Clinical Trials Unit, University of East Anglia, Norwich, UK; Norwich Medical School, University of East Anglia, Norwich, UK; Norwich Clinical Trials Unit, University of East Anglia, Norwich, UK; Norwich Clinical Trials Unit, University of East Anglia, Norwich, UK; Department of Respiratory Medicine, University Hospitals of Leicester NHS Trusts, Leicester, UK; Usher Institute, University of Edinburgh, Edinburgh, UK; Norwich Medical School, University of East Anglia, Norwich, UK

## Abstract

**Introduction:**

Screening for smoking when people interact with healthcare services and referral of those who smoke to stop smoking services (SSSs) is a key component of efforts to tackle tobacco use. However, little is known about what happens after someone is referred or signposted to SSSs.

**Methods:**

As part of the Cessation of Smoking Trial in the Emergency Department (NCT04854616), those randomized to intervention (*n* = 505) were referred to local SSSs (along with receiving brief advice and an e-cigarette starter kit) and those randomized to control (*n* = 502) were given contact details for the same services (signposted). SSS engagement data were collected: (1) directly from participants and (2) from SSS, additional qualitative data came from 33 participant interviews.

**Results:**

Engagement with SSSs was very low. 3.2% (*n* = 16) of those in the intervention group and 2.4% (*n* = 12) in the control group reported attending a one-to-one support session. From SSS data, engagement was also low with 8.9% (*n* = 43) of those referred engaging and 3.1% (*n* = 15) going on to quit with SSS support. The majority of the 24 intervention participants interviewed did not recall being contacted by an SSS.

**Conclusions:**

Referral or signposting to SSSs within an Emergency Department-based trial resulted in very low levels of engagement. Barriers to engagement identified included participants not being contacted by SSSs and the support offered not meeting their needs.

**Implications:**

Referral or signposting of those who smoke to SSSs from the Emergency Department resulted in low rates of engagement in this large multicenter randomized controlled trial. To better support those who smoke, it may be more effective for smoking cessation advice to be offered “in the moment” within clinical settings, and follow-up to be proactively offered rather than relying on people being motivated to contact the services themselves or engaging when contacted.

## Introduction

Smoking is a leading cause of death and disease and a significant cause of health inequality.^1,2^ A key part of the approach to addressing tobacco use in many countries is screening users of healthcare services, giving brief advice to those who smoke, and then referring to community stop smoking services (SSSs). In the United Kingdom, this approach has been recommended by The National Institute for Health and Care Excellence,^3^ Public Health England,^4^ and the NHS Long Term Plan.^5^ However, relatively little is known about what happens when patients are referred or signposted to SSSs.^6^

UK local authority SSSs offer a range of support to help people quit, including one-to-one appointments, group counseling, prescriptions for Nicotine Replacement Therapy (NRT), agonist medications, and, in some cases, e-cigarettes are offered or their use is supported as part of a quit attempt.

Those who engage with SSSs are three times more likely to succeed in quitting than those who attempt to do so without any aid.^7^ Despite the proven effectiveness of such services, their use in the United Kingdom has fallen for 8 consecutive years, from 816 444 people in 2011/2012 to 178 198 in 2021/2022.^8^ In part, this may be due to funding to local SSSs being cut by 30% between 2014 and 2018.^9^

In this study, we define “engagement” as taking up a referral to an SSS either by attending an appointment, accepting a prescription, or setting a quit date. The general model used by SSSs is to encourage people to set a quit date after which they will attempt not to smoke tobacco. Data are regularly published on the proportion of people who successfully quit having set a quit date with SSS^10^; however, data are not available for the number of those who are referred to SSSs who go on to set a quit date (i.e. engagement rates). This paper adopts a mixed-methods approach to demonstrate both levels of engagement and potential barriers to engagement which would not be possible with quantitative data alone.

A recent systematic review concluded that the proactive referral of smokers to smoking cessation programs by healthcare staff is effective at increasing enrollment.^11^ The review identified five studies that investigated proactive e-referral.^11^ These five studies found very different rates of engagement with SSSs for those actively referred 7.8%,^12^ 10.3%,^13^ 14.7%,^14^ 29.5%,^15^ and 31%.^16^ This variation may be explained by differences in the definition of engagement used, with both trials that found an engagement rate of over 20% using the definition of participants having registered with an online system, regardless of whether they had gone on to use the system or receive support.

We aimed to explore engagement with SSSs after referral or signposting from the Emergency Department (EDs) using a mixed-methods approach, within the context of a randomized controlled trial.

## Methods

The Cessation of Smoking Trial in the ED (ClinicalTrials.gov NCT04854616) is a two-arm, multicenter, individually randomized controlled trial. Full details and results can be found in the published protocol and results.^17,18^ Participants randomized to the intervention group were referred by stop smoking advisors in the ED to the local SSSs via the standard electronic referral route used at the NHS site as well as receiving brief advice and an e-cigarette starter kit. Referral triggered proactive telephone contact by the SSSs. Control participants were given a card with contact details for the local SSSs.

Data for this study have been collected via three sources: (1) participants self-reporting service usage and smoking data, (2) information supplied by the SSSs, and (3) qualitative data collected via participant interviews.

Written informed consent was given for both participation in the trial and in the qualitative substudy. On completion of the 6-month follow-up questionnaires, participants received a £30 shopping voucher for taking part. A further £30 voucher was offered to participants who reported being smoke free for providing a CO reading. Participants were, however, unaware they would be offered the additional £30 when completing follow-up questionnaires to avoid it acting as an incentive. Participants received £20 voucher for taking part in the qualitative interviews.

### Participant-Reported Data

At baseline and 6-month follow-up, all participants were asked about their use of SSSs in the past 6 months (“In the past 6 months, have you: Attended a group session with someone from a Smoking Cessation Service [in-person or remotely], Attended a one-to-one session with someone from a Smoking Cessation Service [in-person or remotely], Telephoned the NHS Smoking Helpline service for advice or support”). At 6 months, all participants were asked whether they smoked. Those who reported abstinence were asked to biochemically verify that abstinence with an exhaled carbon monoxide test (with a reading of <8 ppm being used to confirm abstinence).

### Stop Smoking Service Data

Participants in the intervention arm were referred to one of six SSSs. These services were asked to provide the following data for participants who were referred to them as part of the COSTED trial: number of referrals received, number of people contacted, the number who engaged, and the number who were recorded as having gone on to quit. Participants in the control arm were not referred, but instead signposted to SSSs; however, engagement data on these participants were not available due to it not being possible to identify these participants from SSS records.

### Qualitative Data

Interviews were undertaken with 34 participants. Purposive sampling was used to recruit a range of genders, ethnicities, randomization group, site of recruitment, and change in smoking habits (quit, reduced tobacco, and no change). The interviews were conducted via telephone or video call, recorded, and transcribed verbatim.

### Analysis

Rates of engagement were explored descriptively and differences in rates of engagement between intervention and control were analyzed using chi-squared tests. Qualitative data relating to SSS engagement were extracted from interview transcripts and analyzed thematically^19^ using the COM-B model as a theoretical framework with themes identified based on frequency and saliency and classified according to the relevant part of the participant journey.

## Results

### Participant-Reported Data

Between January and August 2022, 1007 participants were randomized (505 to intervention and 502 to control). At 6 months follow-up, 366 (72.5%) participants in the intervention group and 325 (64.7%) in the control group reported their smoking status at 6 months, and 330 (65.3%) in the intervention group and 306 (61.0%) reported whether they had engaged with a SSS.

Overall, the number of participants reporting attending a group SSS session since recruitment was 1.1% (*n* = 11), attending a one-to-one SSS session was 2.8% (*n* = 28), and contacting an NHS smoking helpline was 1.9% (*n* = 19). Table 1 presents the data by randomization group. There was no significant difference between intervention and control.

**Table 1.** Number of Participants Reporting Accessing SSS by Randomized Group at 6-Month Follow-Up

		Intervention (% of those randomized)	Control (% of those randomized)	Chi-square statistic	*p*-Value for difference
Number randomized		505	502		
Number reporting having attended a group session with someone from smoking cessation service	Yes	3 (0.6%)	8 (1.6%)	2.7165	.0993
	No	327 (64.7%)	298 (59.4%)		
Number reporting having attended a one-to-one session with someone from a Smoking Cessation Service	Yes	16 (3.2%)	12 (2.4%)	0.3241	.5691
	No	314 (62.2%)	294 (58.6%)		
Number reporting having telephoned the NHS Smoking Helpline service for advice or support	Yes	10 (2.0%)	9 (1.8%)	0.0044	.9474
	No	320 (63.3%)	297 (59.2%)		

### Stop Smoking Services Data

Of the six SSSs contacted, five provided data. Of 461 participants in the intervention group who were referred to the five services that provided data, there were 316 referrals received (68.5%), 279 (60.5%) were contacted by the service, 43 people engaged with the service (9.3%), and 15 (3.3%) had quit as per the SSS follow-up. This compares to the 124 (25.7%) in the intervention group who reported continuous smoking abstinence at 6 months and 36 (7.5%) who had biochemically validated continuous smoking abstinence at trial follow-up. Data for each site is available in Table S1.

Two services provided reasons for not contacting participants, 32 were not able to be contacted, and 5 were out of the area.

### Qualitative Data

Of the 34 participants interviewed, 33 provided usable data on SSS engagement (one recording was inaudible) of whom 24 were in the intervention group and 9 in the control. Themes and illustrative quotes are included in Table S2. Participant interview sample characteristics are available in Table S3.

### Contact

The anticipated method of contact for the intervention participants was that they would receive a phone call from the SSS with an offer of support, and for control participants, they would contact the phone number on the written material themselves to seek support.

Of the 24 intervention participants who were interviewed, 11 recalled being contacted by the SSS and the remaining 13 did not recall receiving any communication from them.

Of the 10 control participants who were signposted to SSSs, 3 went on to contact them.

Both figures are probably higher than the whole sample because participants who had quit smoking were purposively sampled.

A common theme from some participants who did not recall any communication was that they felt they would have benefitted from support from the SSS.

The low levels of contact potentially provide an explanation for the low levels of engagement seen in the quantitative data.

### Engagement

Of the 11 intervention participants who recalled contact by the SSS, 2 participants engaged and 9 did not.

Of the two who engaged, one participant found them helpful. The other participant engaged and was sent NRT but relapsed into smoking. They reported the phone calls were brief and did not contain any behavioral support.

All three in the control group who contacted the SSS went on to engage, one was given an e-cigarette and managed to quit but then relapsed.

One participant contacted the SSS but said this was unrelated to taking part in the study. The third decided to quit smoking and contacted their GP who advised them to contact the SSS. They struggled to get an appointment with the SSS and also struggled to access NRT. They also reported the approach did not align with how they wanted to quit.

## Discussion

This study found that referral or signposting to SSSs from the ED within the COSTED trial rarely resulted in engagement. Barriers to engagement included lack of contact by SSSs, the support offered not being flexible enough to meet participant’s needs, and offering interventions that were not acceptable to participants. This potentially has implications for service commissioning and delivery.

Relatively few of those referred to SSSs went on to engage (less than 3% for all types of services based on participant data and 11% based on SSS data). The 11% rate based on SSS data is in keeping with previous trials where engagement was classified as receiving stop smoking advice or treatment. Based on the qualitative and SSS data, a possible reason for this is the low rates of the SSSs successfully contacting referred participants (60% based on SSS data and 42% of the qualitative sample). This may be due to SSSs trying to contact participants and being unable to get through. Further possible reasons based on the qualitative data are the services not being flexible enough to meet people’s needs and not offering the types of cessation methods that people wished to use.

There was a large disparity between the number of participants who quit smoking while supported by the SSSs (*n* = 15) and the number who self-reported 6-month continuous abstinence (*n* = 117) implying the vast majority of those who quit within the context of this trial did so without input from the SSSs.

Surprisingly (and contrary to previous studies^11^), there was no difference in engagement with the services between those who were actively referred to the SSSs and those who were merely signposted to the SSSs by providing contact details, although numbers were very small in both groups. A possible reason for this is the relatively low contact rate of those referred (discussed above) or a reason given by some participants in the qualitative interviews is that those in the intervention group (who were referred) also received behavioral support, and an e-cigarette at enrollment, therefore, may not have felt they needed ongoing support from the SSS. Whether someone is referred to a SSS or signposted has not been previously considered as impacting the effectiveness of SSSs.^20^

The low rates of engagement after referral or signpost indicate that delivering interventions in healthcare settings opportunistically may be more effective than relying on referral to external services. There is evidence that smoking cessation interventions delivered in EDs are effective at achieving abstinence.^18^ However, there needs to be sufficient resources allocated to this so as not to burden existing staff.

The strengths of this study are that a large number of people were randomized to either be referred or signposted to SSSs; we were able to triangulate responses using the three data sources; and we collected both self-reported and biochemically verified quit rates. It also benefits from having a group that received an active intervention (e-cigarette and brief advice) and a group that only received signposting. Generalizability is improved by the fact participants were referred to six different SSSs across the United Kingdom. This study collected real-world data in that participants were identified in a healthcare setting (the ED) and referred or signposted to the local service, therefore, reflecting what might happen if such an intervention was implemented in practice. The inclusion of qualitative data allows the identification of barriers to engagement and therefore potential targets for improvements.

The limitations of the study include missing data, with a third of participants not reporting their use of SSSs, differences in how SSSs reported data limiting comparability, one service not providing data and there being some missing data. The lack of an accepted definition of “engagement” made comparison difficult. The qualitative data were purposively sampled, so some groups are overrepresented. We were also collecting data from participants 6 months after randomization, so it is possible they forgot the contact from the SSS or would not have been aware if contact attempts were made but not successful (although this was mitigated by contacting the SSSs as well). The fact those who were referred also received brief advice and an e-cigarette also limits direct comparison between the groups.

## Conclusions

Referral or signposting of those who smoke to SSSs from the ED resulted in low rates of engagement in this large multicenter randomized controlled trial. To better support those who smoke it may be more effective for smoking cessation advice to be offered “in the moment” within clinical settings, and follow-up to be proactively offered rather than relying on people being motivated to contact the services themselves or engaging when contacted. These findings have implications for policy-makers looking to maximize the reach and effectiveness of services.

## Supplementary material

Supplementary material is available at *Nicotine and Tobacco Research* online.

ntae159_suppl_Supplementary_Tables_1-3

## Data Availability

The protocol, consent form, statistical analysis plan, medical ethics committee approvals, training materials, and other relevant study materials are available online at https://osf.io/8hbne/. Deidentified participant data will be made available upon reasonable request.
