# Supplementary material for: Engagement With Stop Smoking Services After Referral or Signposting: A Mixed-Methods Study
Source: Nicotine Tob Res. 2024 Jul 3;27(2):360–3. doi: 10.1093/ntr/ntae159 (PMC11750743; doi:10.1093/ntr/ntae159)
Supplement: ntae159_suppl_Supplementary_Tables_1-3 [file ntae159_suppl_supplementary_tables_1-3.docx]

Supplementary table 1: data on those randomised to the intervention group from sites, stop smoking services and participants.

|  |  | | Site 1  (% of those randomised) | Site 2  (% of those randomised) | Site 3  (% of those randomised) | Site 4  (% of those randomised) | Site 5  (% of those randomised) | Site 6  (% of those randomised) | Total- excluding site 6 (% of those randomised) |
| --- | --- | --- | --- | --- | --- | --- | --- | --- | --- |
| Data from sites | Randomised to intervention | | 207 | 89 | 54 | 81 | 51 | 23 | 482 |
|  | Referred | | 195 (98.0%) | 88 (100.0%) | 54 (100.0%) | 74 (100.0%) | 50 (100.0%) | 22 (95.7%) | 461 (95.6%) |
| Data from stop smoking services | Referral received | | 182 (87.9%) | 21 (23.6%) | 31 (57.4%) | 32 (39.5%) | 50 (98.0%) | - | 316 (65.6%) |
|  | Contacted | | 151 (72.9%) | 42 (47.2%) | 24 (44.4%) | 32 (39.5%) | 30 (98.0%) | - | 279 (57.9%) |
|  | Engaged | | 1 (0.5%) | - | 20 (37.0%) | 5 (6.2%) | 17 (33.3%) | - | 43 (8.9%) |
|  | Quit according to service | | 0 (0.0%) | 4 (4.5%) | 6 (11.1%) | 2 (2.5%) | 3 (5.9%) | - | 15 (3.1%) |
| Data from participants | Self-reported 6 months continuous abstinence | | 53 (25.6%) | 23 (25.8%) | 13 (24.1%) | 18 (22.2%) | 17 (33.3%) | 5 (21.7%) | 124 (25.7%) |
|  | Biochemically validated | | 16 (7.7%) | 5 (5.6%) | 6 (11.1%) | 5 (6.2%) | 4 (7.8%) | 2 (8.7%) | 36 (7.5%) |
|  | Attended a SSS group session | Yes | 1 (0.5%) | 0 (0.0%) | 1 (1.9%) | 1 (1.2%) | 0 (0.0%) | 0 (0.0%) | 3 (0.6%) |
|  |  | No | 147 (71.0%) | 56 (62.9%) | 31 (57.4%) | 41 (50.6%) | 38 (74.5%) | 14 (60.9%) | 313 (64.9%) |
|  | Attended a SSS one-to-one session | Yes | 4 (1.9%) | 1 (1.1%) | 4 (7.4%) | 4 (4.9%) | 3 (5.9%) | 0 (0.0%) | 16 (3.3%) |
|  |  | No | 144 (69.6%) | 55 (61.8%) | 28 (51.9%) | 38 (46.9%) | 35 (68.6%) | 14 (60.9%) | 300 (62.2%) |
|  | Telephoned the NHS Smoking Helpline | Yes | 5 (2.4%) | 0 (0.0%) | 0 (0.0%) | 3 (3.7%) | 2 (3.9%) | 0 (0.0%) | 10 (2.1%) |
|  |  | No | 143 (69.0%) | 56 (62.9%) | 32 (59.3%) | 39 (48.1%) | 36 (70.6%) | 14 (60.9%) | 306 (63.5%) |

**Supplementary table 2: Qualitative data themes and illustrative quotes**

| Theme category | Theme | Illustrative quote |
| --- | --- | --- |
| Contact | Would have taken up support if it had been offered | *“I think I would [have taken up support] because I would have felt it was a bit of extra support” (*Male, 50-59, quit) |
| Engagement | Did not have time to take up support | *“I just don’t think I had the time…I just don't think I would be very good at committing to it.”* (Female, 20-29, smoking) |
|  | Felt being in the trial was enough | *“I’d have probably told them I was doing OK with the vaping”* (Male, 50-59, tobacco reducer)  *“I had already started vaping… I've already started the support. I would want to finish this course [COSTED] out before I go on to something else.”* |
|  | Did an initial phone call but felt it was not for them | *“The stop smoking team from the NHS phoned me…and at the time I was in a difficult place. But she was very supportive… she didn’t pressure me, she just told me to phone up if I want to and that kind of thing. I did genuinely think that I would give it a go but how things worked out, I just didn’t manage to.” (*Female, 50-59, smoking) |
|  | Had already quit | *“[Having already quit] It was a very light and brief conversation, which probably lasted for about three to five-minutes.”* (Female, 60-69, quit) |
|  | Did not want to quit at time contacted | *“I think I got a text notification through saying with regards to the smoking thing, that if I would be interested to follow up, but at that point in time…I was still happily smoking so I didn't respond, so I didn't arrange the appointment.”* (Male, 20-29, quit) |
|  | Wanted to do it on their own | *“I was contacted but if I'm honest, I didn't engage with them. I tried to do it on my own.” (*Female, 20-29, tobacco reducer) |
|  | Declined because did not want NRT | *“They had offered me the patches, but now my nan had the patches and they made my nan really ill. Now I know it affects people in different ways but I didn’t want to take that risk. I said I thought I'll be OK. Luckily, I said that because I am. I’ve smashed it. Yeah, I denied the support groups and the patches.”* (Female, 20-29, quit)  *“Well, I did, I took part in the phone call. I didn't want to do the…you know, I didn't want any of the medication [NRT]”* (Female, 40-49. tobacco reducer) |
| Experience after engagement | Found the service helpful | *“She wasn't pushing you and, she was really supportive. But I just seemed to engage with her very easily. Whether it makes a difference that you've got somebody that isn't preaching to you but more supportive” (Female, 50-59, quit)* |
|  | Found the phone calls brief and did not contain behavioural support | *“It didn’t have any effect….[I would have liked] more encouragement on actually trying to quit. A bit more information on stress handling techniques. Not just, “here's your stuff [NRT]. It will be with you in so many days. Try not to smoke until then.” (Female, 30-39, tobacco reducer)* |
|  | Appointment times not convenient | *“I think I contacted them and there was a bit of confusion to start with. I haven't progressed any further with it….Basically, because of my shifts. I mean I start work at half three, and I finish at half past midnight. my mornings are spent on resting from the night before.”* |
|  | Struggled to get an appointment with the SSS | *“They're an absolute pain in the backside to get an appointment with. The appointment times just didn't fit in, obviously having children at school and it was just such a way, I actually ended up, and I quote, saying to them “you’re supposed to be helping people to give up. No wonder nobody quits.” (*Male, 50-59, tobacco reducer) |
|  | SSS approach not aligning with how they wanted to quit. | *“But the lady when she spoke to me, she was like, “OK, well, if this is what you're going to do, you need to make yourself a quit date. You need to get rid of every single tobacco item out of your house.” And I remember saying to her, “well, I don't want to do it like that, because I don’t want to feel like I can’t smoke if I want to. In my head, if it was in the house and I could get to it, then I would probably be less likely to crave it. She was like, “well, I think you're wrong, but do it your way.” (*Female, 30-39, quit) |

**Supplementary table 3: Participant interview sample characteristics**

|  | Trial participants (interview subsample n=34) |
| --- | --- |
| **Site**  Site 1  Site 2  Site 3  Site 4  Site 5  Site 6 | 12  6  6  3  5  2 |
| **Sample group**  Intervention - Quit  Intervention - Harm reduction (reduced cigarettes per day by at least 50%)  Intervention - No/ limited change  Usual Care | 7  12  5  10 |
| **Gender**  Male  Female | 20  14 |
| **Age (years)**  Mean (range)  20-29  30-39  40-49  50-59  60+ | 44 (20-70)  6  8  5  10  5 |
| **Ethnicity**  Asian Bangladeshi  Asian Other  Black British  Black Caribbean  White British  White Eastern European  White Irish  White Other | 2  1  1  2  23  2  2  1 |
| **Employment status**  Employed full-time  Employed part-time  Full-time carer (e.g. of children or other family members)  Retired  Self-employed or freelance  Unable to work due to sickness or disability  Unemployed and looking for work | 15  4  1  2  6  4  2 |
